# Antagonistic genetic correlations for milking traits within the genome of dairy cattle

**DOI:** 10.1371/journal.pone.0175105

**Published:** 2017-04-05

**Authors:** Olivier Gervais, Ricardo Pong-Wong, Pau Navarro, Chris S. Haley, Yoshitaka Nagamine

**Affiliations:** 1 Kyoto University, Graduate School of Informatics, Kyoto Japan; 2 The Roslin Institute and R(D)SVS, University of Edinburgh, Midlothian, United Kingdom; 3 MRC Human Genetics Unit, MRC Institute of Genetics and Molecular Medicine, University of Edinburgh, Western General Hospital, Edinburgh, United Kingdom; 4 Nihon University, Bio-resource Science, Fujisawa, Japan; University of Bonn, GERMANY

## Abstract

Genome-wide association studies can be applied to identify useful SNPs associated with complex traits. Furthermore, regional genomic mapping can be used to estimate regional variance and clarify the genomic relationships within and outside regions but has not previously been applied to milk traits in cattle. We applied both single SNP analysis and regional genomic mapping to investigate SNPs or regions associated with milk yield traits in dairy cattle. The de-regressed breeding values of three traits, total yield (kg) of milk (**MLK**), fat (**FAT**), and protein (**PRT**) in 305 days, from 2,590 Holstein sires in Japan were analyzed. All sires were genotyped with 40,646 single-nucleotide polymorphism (**SNP**) markers. A genome-wide significant region (*P* < 0.01) common to all three traits was identified by regional genomic mapping on chromosome (**BTA**) 14. In contrast, single SNP analysis identified significant SNPs only for MLK and FAT *(P* < 0.01), but not PRT in the same region. Regional genomic mapping revealed an additional significant region (*P* < 0.01) for FAT on BTA5 that was not identified by single SNP analysis. The additive whole-genomic effects estimated in the regional genomic mapping analysis for the three traits were positively correlated with one another (0.830–0.924). However, the regional genomic effects obtained by using a window size of 20 SNPs for FAT on BTA14 were negatively correlated (*P* < 0.01) with the regional genomic effect for MLK (–0.940) and PRT (–0.878). The BTA14 regional effect for FAT also showed significant negative correlations (*P* < 0.01) with the whole genomic effects for MLK (–0.153), FAT (–0.172), and PRT (–0.181). These negative genomic correlations between loci are consistent with the negative linkage disequilibrium expected for traits under directional selection. Such antagonistic correlations may hamper the fixation of the FAT increasing alleles on BTA14. In summary, regional genomic mapping found more regions associated with milk production traits than did single SNP analysis. In addition, the existence of non-zero covariances between regional and whole genomic effects may influence the detection of regional effects, and antagonistic correlations could hamper the fixation of major genes under intensive selection.

## Introduction

The information provided by the numerous SNPs mapped on the whole genome gives us a powerful tool that can be used in breeding to improve complex traits in livestock populations. Genome-wide association studies (GWAS) can be applied to identify useful SNPs associated with complex traits [1, 2]. Furthermore, Meuwissen et al. [3] introduced the idea of using all SNPs together to estimate their total additive genotypic values. The genetic markers distributed throughout the entire genome can be used to construct a genomic relationship matrix between individuals that can then be used in mixed model equations instead of Henderson’s A matrix, a relationship matrix that is based solely on pedigree information [4, 5]. In addition, Nagamine et al. [6] proposed a regional genomic relationship or regional heritability mapping that typically uses 10 to 100 sequential SNPs to detect additive regional genomic variances and their effects. This method has been applied to animal and human data to find quantitative trait loci (QTLs) complementing the GWAS approach [7–14]. Conventional methods in animal breeding that use only pedigree information assume that many undetectable polygenes control complex traits, and the properties of effective regions that harbor loci with detectable effects have not been thoroughly investigated. The additional information afforded by a region that is linked to a QTL can reveal relationships between genes within and between traits.

Recent progress in GWAS has led to the discovery of individual genes that exert substantial effects on traits of economic importance in domestic animals. *DGAT1*, for example, is a well-studied gene with substantial effects on dairy traits in cattle [2, 15]. Alleles of *DGAT1* are segregating rather than being fixed for the single most beneficial genotype, their segregation being the reason why we can detect their effect and position. It is interesting to consider why alleles of such large effect can remain segregating, even after long and intense selection [2, 15, 16]. Applying a regional genomic mapping method using regional and whole genomic information may yield new insights into the improvement of complex traits in farm animals and the genetic architecture of traits under selection.

Our objectives here were to use both single SNP analysis and the regional genomic mapping method to investigate regions with large effects on milk production traits. The ability to evaluate the relationships between whole and regional genomic effects within and between traits might reveal why genes with large effects remain segregating in livestock even after intense selection. As far as we know, this study is the first to have investigated the genomic relationships within and outside regions with large effects on the traits under selection.

## Materials and methods

### Data

We used the de-regressed breeding values of three traits—total yield (kg) of milk (MLK), fat (FAT), and protein (PRT) in 305 days—from 2590 Holstein sires in Japan. Average phenotypic values of daughter cows were approximately 9226 kg for MLK, 364 kg for FAT, and 301 kg for PRT. The sires had been evaluated by using the milking records of more than 50 daughters each, and the results of these evaluations were obtained from the National Dairy Herd Improvement Program of Japan. DNA samples isolated from semen were used for genotyping all sires with the Illumina Bovine 50K beadchip (Illumina, San Diego, CA, USA) SNP array. This array was constructed with 54001 SNPs distributed across the whole genome with an average SNP spacing of 51.5 kb and a median spacing of 37.3kb. All procedures, for example amplifying and hybridizing, were carried out according to the Illumina Infinium assay protocol (Illumina, San Diego, CA, USA) and have been detailed previously [17]. We used the following criteria for marker section: an SNP was removed when either the minor allele frequency (MAF) was less than 1%, or the call rate was less than 95%, or the Hardy–Weinberg equilibrium test had a *P* value lower than 10^−6^. Finally, 40646 SNP markers (Table 1) were used.

**Table 1:** Numbers of SNPs on the chromosomes (BTA) used in the analysis.

	Number of SNPs
BTA 1	2630
BTA 2	2126
BTA 3	1999
BTA 4	1967
BTA 5	1714
BTA 6	2002
BTA 7	1749
BTA 8	1860
BTA 9	1621
BTA 10	1699
BTA 11	1762
BTA 12	1344
BTA 13	1391
BTA 14	1418
BTA 15	1342
BTA 16	1278
BTA 17	1262
BTA 18	1060
BTA 19	1086
BTA 20	1268
BTA 21	1113
BTA 22	1013
BTA 23	868
BTA 24	1003
BTA 25	796
BTA 26	873
BTA 27	786
BTA 28	771
BTA 29	845
Total	40646

### Ethics statement

Standard practices of animal care according to the notification of the Japanese Ministry of Environment (2013; 85) were applied to the animals used. The sires, and their daughters from which we collected the milking records, were not subject to any experimental procedures, and all data were collected with the owners’ permission as part of normal husbandry practices and according to Japanese regulations; hence ethics committee approval was not necessary for this study. The sires were owned and managed by four government-approved insemination organizations in Japan. The SNP data were collected from semen straws provided as part of the regular semen collection process by staff of the insemination stations.

### Mixed model using whole genomic and regional genomic relationship matrices

We used a model in which the additive genomic effect was divided into two parts, namely regional genomic and whole genomic effects. The whole genomic polygenic value was estimated by using all SNPs to construct the whole genomic relationship matrix. The regional genomic polygenic value was obtained from a regional genomic relationship matrix that used 100 (or 20 or 10) adjacent SNPs from each region. The genomic kinship f_ij_ between individuals i and j, using identity by state (IBS), was used, such that:

$$f_{ij}=\frac{1}{n}\sum_{k=1}^{n}\left(\frac{\left(g_{ik}-p_{k})(g_{jk}-p_{k}\right)}{p_{k}(1-p_{k})}\right),$$

where g_ik_ (g_jk_) is the genotype of the i-th (j-th) individual at the k-th SNP (coded 0, 0.5 and 1 for rare-allele homozygotes, heterozygotes and common homozygotes, respectively) [18, 19]. The frequency p_ij_ is for the major allele and n is the number of SNPs. For relationship matrices in mixed model equations, 2f_ij_ is used for the off-diagonal elements between individuals, and the diagonal elements are 1 plus the inbreeding coefficient [18]. A region was called a window and each window overlapped by 50% of SNPs with the previous region. A regional relationship matrix with 100 SNPs (window size, 100), for example, was shifted every 50 SNPs to make the next relationship matrix. Therefore, as an example, the first, second and third regional matrices used from the 1st to 100th, 51st to 150th, and 101th to 200th SNPs, respectively. In total, 798 windows (size, 100) were tested across chromosomes. We used window sizes of 100, 20, and 10, which had been applied in a previous study [6] in which appropriate sizes were discussed.

We followed the two-step method described by George et al. [20] for variance component analysis using ASReml (VSN International, 2002). The mixed model is as follows:

y=1μ+Zu+Zw+e

$$Var(u)=G{\sigma^{2}}_{u},\;Var(w)=Q{\sigma^{2}}_{w},\;Var(e)=I{\sigma^{2}}_{e},$$

where μ is the mean, 1 is a vector, **y** is a vector that represents the de-regressed breeding values, and **Z** is the design matrix for random effects. The remaining vectors are **u**, which is the additive whole genomic effect; **w**, the additive regional genomic effect, and **e**, the residual. **G** and **I** are whole genomic relationship matrices using all SNPs for the whole genomic value and an identity matrix for residuals, respectively. **Q** is the regional genomic relationship matrix obtained by using 100 or fewer SNPs for the regional genomic value. Whole genomic values, regional genomic values, and residuals are random effects, and their variances are σ_u_^2^, σ_w_^2^, and σ_e_^2^, respectively. The details of the regional genomic method have been described previously [6].

### Testing for significant region

To test for the presence of regional variance against the null hypothesis (no regional variance) at a test region (window), the likelihood ratio (LR) test statistic LRT = –2ln(L_0_/L_1_) was calculated, where L_0_ and L_1_ represent the respective likelihood values under the hypothesis of either the absence (H_0_) or presence (H_1_) of regional variance. The LRT is assumed to be drawn from a distribution under the null hypothesis that is a mixture of chi-square distribution with 1 degree of freedom and zero (Visscher [21]) with Bonferroni correction. Approximately 800, 4,000, and 8000 windows were tested for window sizes of 100, 20, and 10 SNPs, respectively. The genome-wide significant thresholds were: LRT (>17.76 for 1%, >14.71 for 5% level) with size 100, LRT (>20.83 for 1%, >17.76 for 5% level) with size 20, and LRT (>22.16 for 1%, >19.08 for 5% level) with size 10.

### Single SNP association analysis

We applied the concept of GRAMMAR (Genome-wide Rapid Association using Mixed Models and Regression) by Aulchenko et al. [22] for single SNP analysis. This method first obtains residuals that are adjusted for whole genomic values and subsequently analyzes the association between these residuals and single SNPs by using rapid least-squares methods. Because we used de-regressed estimated breeding values as our observations, residuals corresponding to the reduction of whole genomic values were applied as the left-hand side in the regression model. F values adjusted by Bonferroni correction for 40646 SNPs were applied to test the regression coefficient.

## Results

### Regional genomic variance and single SNP analysis

**Fig 1:**
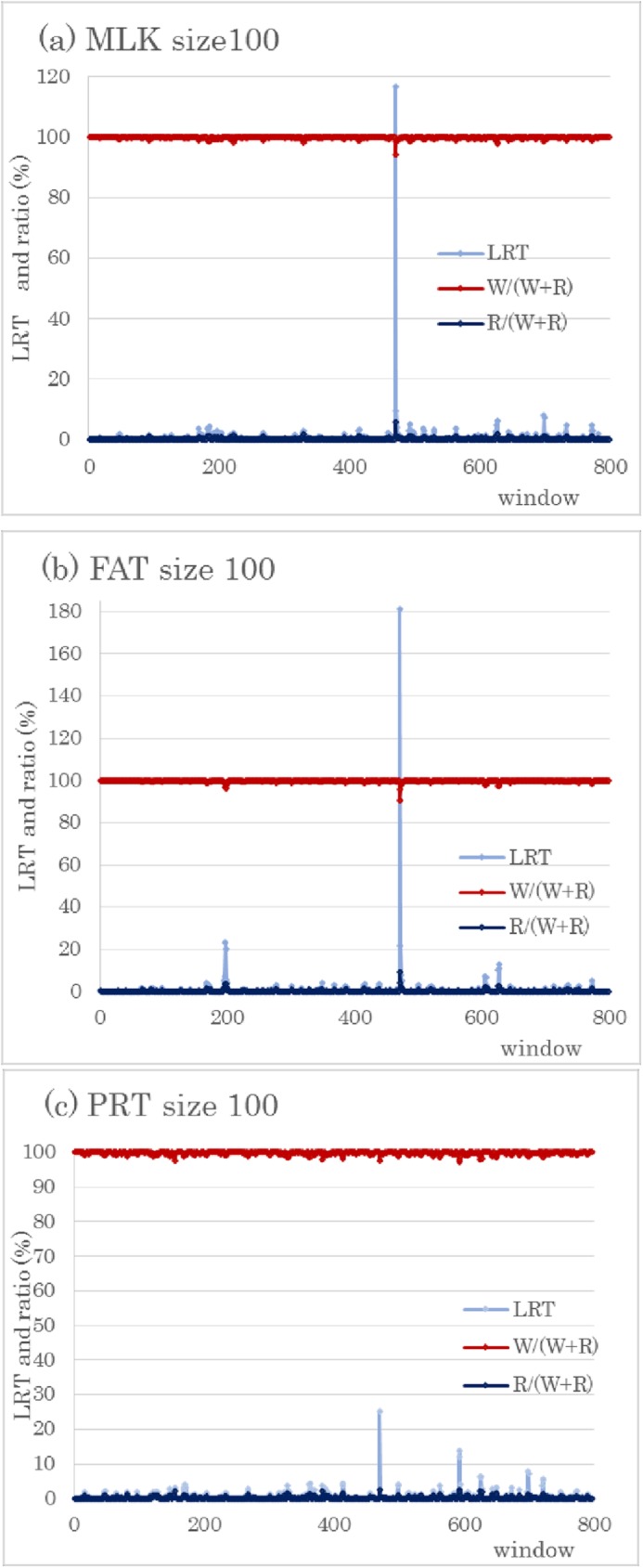
LRT and percentages of regional (R) and whole genomic variances (W) in total genomic variances (W+R) for three yield traits: (a) milk (MLK), (b) fat (FAT), and (c) protein yield (PRT). The vertical axis indicates the likelihood ratio test (LRT) and ratio (%), and the horizontal axis shows the window number across the genome. In total, 798 windows, each using 100 SNPs, were examined.

**Fig 2:**
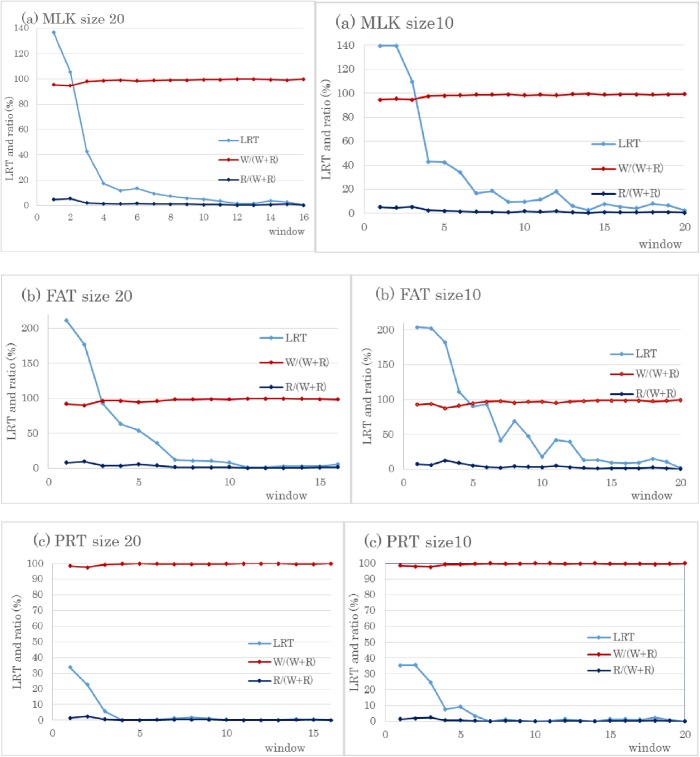
LRT and percentages of regional (R) and whole genomic variances (W) in total genomic variances (W+R) using 20 or 10 SNPs for BTA14. The vertical axis indicates the likelihood ratio test (LRT) and ratio (%), and the horizontal axis is the window number at the end of BTA14. (a) milk (MLK), (b) fat (FAT), and (c) protein yield (PRT); size 20 and size 10 are window sizes using 20 SNPs and 10 SNPs, respectively.

We estimated regional genomic variance by using 100 or fewer SNPs to obtain likelihood ratio test values (LRT). The first window (size 100) on BTA14 had the largest LRT for MLK (116.8), FAT (181.3), and PRT (25.2) (Fig 1). Focusing on the significant region on BTA14, we used 20 SNPs for regional genomic matrices. The first window (size 20) on BTA14 displayed the highest LRTs for all three traits: MLK, 136.8; FAT, 210.9; and PRT, 33.7 (Fig 2). After a window of size 10 was applied, the LRTs from the first window for the three traits were: MLK, 139.6; FAT, 204.2; and PRT, 35.4. Therefore, the largest genome-wide–log^10^ *P*-values were 27.9 (LRT, 139.6) for MLK and 5.0 (LRT, 35.4) for PRT by using window size 10; for FAT the value was 43.8 (LRT, 210.9) by using window size 20. A significant region common to all three traits was found only on BTA14; however, BTA 5 had a significant region with LRT 23.3 (*P* < 0.01) for FAT when the window size was 100. This value increased to LRT 23.7 with a window size of 20. In addition, BTA18 had a relatively large LRT 13.7 for PRT using window size 100, and it increased to 15.6 at a window size 20. The genomic variances for the three traits from whole genomic values and regional genomic values (first window, size 20) on BTA14 are given in Table 2. The percentages of regional genomic variance relative to the total genomic variance (whole and regional genomic variances) are also shown. Regions with larger LRT values had relatively large regional variances.

**Table 2:** Whole and regional genomic variances (Var), SNP names, and regional variance ratios, with LRT, on BTA14, BTA5, and BTA18.

	Trait ^1)^	Whole genomic Var	Regional genomic Var^2)^	First and last SNP names (pb)^3)^	R/(W+R) %^4)^	LRT
BTA14	MLK	5009.6	240.9	Hapmap30381-BTC-005750 (1463676)	4.6	136.8
				Hapmap30086-BTC-002066 (2524432)		
	FAT	485.6	42.7		8.1	210.9
	PRT	393.2	6		1.5	33.7
BTA5	FAT	543.9	27.2	Hapmap48620-BTA-113306 (91799509)	4.8	23.7
				Hapmap36414-SCAFFOLD150043_20489 (93567346)		
BTA18	PRT	399.7	6.1	BTA-97501-no-rs (57565406)	1.5	15.6
				ARS-BFGL-NGS-118325 (60103953)		

1) MLK (milk), FAT(fat), and PRT(protein) yield in kg.

2) Regional variances were estimated by using a significant window (size 20) from each chromosome.

3) First and last SNP names and position of the window.

4) Percentages of regional genomic variance in the total genomic variance (whole and regional variances).

By using a single SNP association method, we found significant SNPs only on BTA14 for MLK (4 SNPs with *P* < 0.01 and 3 SNPs with *P* < 0.05) and for FAT (11 SNPs with *P* < 0.01 and 4 SNPs with *P* < 0.05) but no significant SNPs for PRT (Table 3). Almost all significant SNPs were located within the first window (size 20) on BTA14. SNPs with relatively large F-values—outside BTA14—were found on BTA5 for FAT and BTA18 for PRT, but they did not exceed the genome-wide level of significance.

**Table 3:** SNPs with the largest F-values by single SNP analysis within significant regions ^1)^.

Trait^2)^	Chromo-some	Consecutive number ^3)^	SNP	Position	F-val	Log_10_ P value	Genome-wide Pr ^4)^
MLK	BTA14	23871	ARS-BFGL-NGS-4939	1801116	68.05	15.60	P<0.01
FAT		23871	ARS-BFGL-NGS-4939	1801116	93.17	20.96	P<0.01
PRT		23871	ARS-BFGL-NGS-4939	1801116	21.37	5.40	ns
FAT	BTA 5	9981	Hapmap33512-BTA-158274	92283403	7.79	2.28	ns
PRT	BTA18	30128	Hapmap40906-BTA-121147	58551307	8.14	2.36	ns

1) Significant regions were estimated by using window size 20.

2) MLK (milk), FAT (fat), and PRT(protein) yields, in kg.

3) Consecutive SNP number in this analysis.

4) Genome-wide significance level was 1% (–log10 *P* > 6.61) or 5% (–log10 *P* > 5.91).

### Correlations between whole and regional genomic effects

Correlations between whole and regional genomic effects by using the first window (size 20) on BTA14 were calculated (Table 4). All correlations between the additive whole genomic effects from the three traits were positive (0.830–0.924). However, the regional genomic effects for FAT were negatively correlated (*P* < 0.01) with the regional genomic effect for MLK (–0.940) and PRT (–0.878), and regional effects for FAT also showed negative correlations (*P* < 0.01) with the whole genomic effect for MLK (–0.153), FAT (–0.172), or PRT (–0.181). The regional genomic effect for FAT (LRT 23.7) at window size 20 on BTA 5 showed almost no correlation (0.07) with the whole genomic effect for FAT. PRT (LRT 15.6) on BTA18 had a moderately positive correlation, 0.29, with the whole genomic effect for PRT.

**Table 4:** Correlations between whole and regional genomic effects for the three traits.

		Whole			Regional		
		MLK	FAT	PRT	MLK	FAT	PRT
Whole genomic effect	MLK	1	0.830	0.924	0.188	-0.153	0.197
	FAT		1	0.892	0.198	-0.172	0.198
	PRT			1	0.225	-0.181	0.234
Regional genomic effect	MLK				1	-0.940	0.978
	FAT					1	-0.878
	PRT						1

Regional genomic effects were estimated from the first window (size 20) on BTA14

MLK (milk), FAT (fat), and PRT (protein) yields are in kg

**Fig 3:**
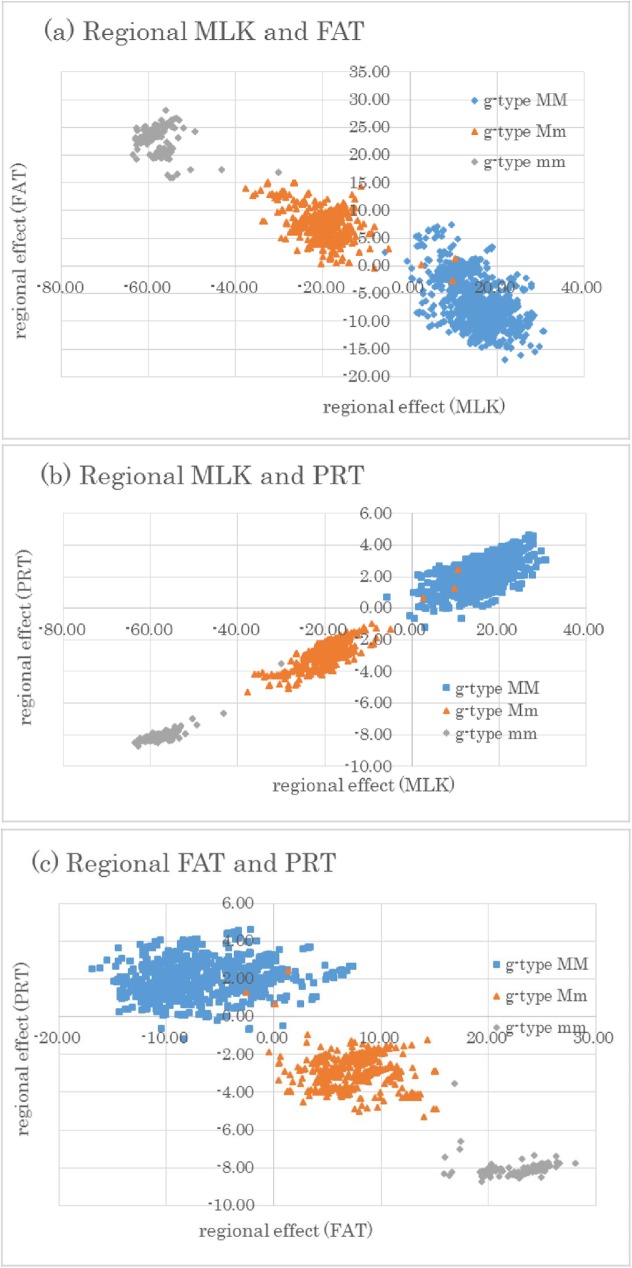
Correlation between regional genomic effects of three traits (MLK, milk; FAT, fat; PRT, protein yield) from the first window using 20 SNPs on BTA14. Individuals were categorized into three genotypes with the most significant SNP (ARS-BFGL-NGS-4939) in this region. Genotypes (g-type) MM, mm, and Mm are major and minor homozygotes and heterozygotes, respectively. The vertical axes are the regional genomic effects of (a) fat and (b, c) protein yield, and the horizontal axes are (a, b) milk and (c) fat yield.

We categorized individual bulls with three genotypes that had the most significant SNP common to the three traits (ARS-BFGL-NGS-4939) from the first window (size 20) on BTA14 and plotted their regional genomic effects to display the correlations between the three traits (Fig 3). Individuals with major homozygotes, minor homozygotes, and heterozygotes of this marker displayed separate distributions between groups. Individuals with higher values of regional effect for FAT showed lower values for MLK and for PRT. Therefore, this highly significant SNP marker appears to have antagonistic pleiotropic effects on fat and the other two traits.

## Discussion

By using a regional genomic mapping approach we identified significant regions (*P* < 0.01) on BTA 14 for all three traits (MLK, FAT, and PRT) and on BTA 5 for FAT, and a relatively large LRT (*P* < 0.1) on BTA 18 for PRT. SNPs from some of these regions have been reported in previous studies using single SNP association approaches (e.g., [2, 23, 24]). Grisart et al. [15] found very strong evidence of a QTL at the end of BTA14 that has a major effect on milk yield. We found the most significant window (size 20) on BTA14 around SNP ARS-BFGL-NGS-4939, which showed the largest F value in Table 3. Wang et al. [2] similarly reported that this marker was the most significant SNP for fat percentage and that it was related to the acyl-CoA:diacylglycerol acyltransferase-encoding gene *DGAT1*. Given these previous reports and our current results, the candidate gene relating to FAT on BTA14 likely is *DGAT1*. Our regional genomic method confirmed the presence of significant regions for the three traits on BTA14, but the single SNP analysis in our data was able to detect effects for MLK and FAT only (Table 3). Specifically, 4 SNPs for MLK and 11 SNPs for FAT achieved significance at the 1% level, but there were no significant SNPs for PRT on BTA14 (*P* > 0.05). Given the previous findings, *DGAT1* affects not only FAT and MLK but also PRT [15]. The single SNP association approach demands that at least one SNP reaches significance. In contrast, the regional genomic method relies not on a single SNP but on related SNPs in the same region. Therefore, its power to detect associated regions may be greater than that of methods based on single SNPs [6, 10].

We found the second-largest LRT for FAT in a window (size 20) on BTA 5. The position (92.6 Mb) of the middle of this region is very close to a previously reported SNP at 92.1 Mb for MLK [24]. Wang et al. [2] also reported a significant SNP for fat percentage at 94.6 Mb on BTA5; the candidate gene was *EPS8*. However, our single SNP analysis did not find any significant SNP in this region.

When Grisart et al. [15] described the major effects of *DGAT1* on milk yield traits, they reported an extremely large proportion of the variance due to the QTL on BTA14 in the total genetic variance. Using a fixed regression model for the substitution effect of two alleles, they reported that the QTL explained more than 27% (= QTL 18% / (QLT 18% + polygene 49%)) of the total genetic variance for MLK and 21% (= QTL 15% / (QTL 15% + polygene 55%)) of that for FAT. However, our regional genomic mapping from the first window (size 20) on BTA14, including the *DGAT1* region, estimated 8.1% for FAT, 4.6% for MLK, and 1.5% for PRT (Table 2). The regions with higher LRT had larger relative sizes in terms of total genetic variance, consistent with the findings of our previous study [6]. Grisart et al. [23] assumed that *DGAT1* had two alleles, but they did not exclude the existence of other, as yet uncharacterized, *DGAT1* alleles that might contribute to genetic variance in milk traits. Regional genomic mapping using simultaneous estimation of both whole and regional genomic variances in mixed models that did not assume a particular number of alleles might have yielded less bias in variance estimation. We also reported that the most notable feature of the regional genomic approach was that, in a simulation study, its power was affected little by the number of SNPs [6]. In contrast, when only two alleles segregated in a region, single SNP analysis was similar in power to regional genomic analysis. When a cluster of functional variants was segregated in a region, the regional genomic approach captured more of the genetic variance segregating in the population, locating loci that otherwise would have remained undetected.

We might estimate more accurate variances and regions by using a regional mapping approach and use these results for genomic selection; however, fixing preferred genes in these regions may be difficult. Correlations between the regional genomic effect for FAT on BTA14 and either the whole genomic effect for FAT or the whole or regional genomic effects of MLK and PRT were negative (Table 4).

Grisart et al. [15, 23] and Thaller et al. [25] similarly reported that positive allelic substitution effects of *DGAT1* for fat yield had negative effects on milk and protein yield. Therefore, these previous results are in agreement with ours. However, a negative correlation between the whole genomic effect and the regional genomic effect for FAT has not yet been reported.

Although it is not possible to reach a definitive conclusion, it is known that the Bulmer [26] effect can cause negative correlations between selected loci. Itoh [27] applied this theory in the selection of multiple traits; he reported that the genetic correlation between traits with higher heritabilities changed faster to an undesirable direction. We estimated the antagonistic correlation between regional genomic effects with larger LRT (which have larger genetic variances) and the whole genomic effect. One result, however, of such an antagonistic correlation of regional effects may be to allow major genes to remain segregating that might otherwise be rapidly fixed in a selected population.

In addition, relationships between a region and the whole genome might cause bias in the estimation of both regional and whole genome variances. If we consider only the whole genomic effect without regional or SNP effects in a model, for example, then negative correlation reduces the apparent whole genomic variance and heritability. In contrast, a positive relationship between a region and whole genome leads to increased genetic variance and heritability.

Considering only the regional effect or SNP effect in a model, a negative correlation with the whole genomic effect can reduce the regional variance and hamper the detection of the effective regions or SNPs. If correlations prove to be a widespread phenomenon across populations and species, this method might produce the interesting consequence that the summed effect of the region and the whole genome differ from the total genetic variance due to the contribution of the covariance. A positive covariance might inflate the total heritability in comparison to the sum of its components, resulting in an apparent “missing heritability” [28] due to the ignored covariance.

Here, we revealed the relationships between the whole and regional genomic effects for some important dairy traits and provided a possible explanation for why genes with large effects remain segregating in livestock even after intense selection. Applying these approaches to the analysis of larger single-locus and regional effects in other livestock likely would be informative. Such analyses may shed light on both trait genetic architecture and selection in these populations.
